# Can hospital audit teams identify case management problems, analyse their causes, identify and implement improvements? A cross-sectional process evaluation of obstetric near-miss case reviews in Benin

**DOI:** 10.1186/1471-2393-12-109

**Published:** 2012-10-11

**Authors:** Matthias Borchert, Sourou Goufodji, Eusèbe Alihonou, Thérèse Delvaux, Jacques Saizonou, Lydie Kanhonou, Véronique Filippi

**Affiliations:** 1 Department of Infectious Disease Epidemiology, London School of Hygiene & Tropical Medicine, London, UK; 2 Institute of Tropical Medicine and International Health, Charité - Universitätsmedizin Berlin, Berlin, Germany; 3 Centre pour la Recherche en Reproduction Humaine et Démographie, Cotonou, Benin; 4 Institute of Tropical Medicine, Antwerp, Belgium

**Keywords:** Obstetrics, Near-miss complications, Quality assurance, Audit, Benin, West Africa

## Abstract

**Background:**

Obstetric near-miss case reviews are being promoted as a quality assurance intervention suitable for hospitals in low income countries. We introduced such reviews in five district, regional and national hospitals in Benin, West Africa. In a cross-sectional study we analysed the extent to which the hospital audit teams were able to identify case management problems (CMPs), analyse their causes, agree on solutions and put these solutions into practice.

**Methods:**

We analysed case summaries, women’s interview transcripts and audit minutes produced by the audit teams for 67 meetings concerning one woman with near-miss complications each. We compared the proportion of CMPs identified by an external assessment team to the number found by the audit teams. For the latter, we described the CMP causes identified, solutions proposed and implemented by the audit teams.

**Results:**

Audit meetings were conducted regularly and were well attended. Audit teams identified half of the 714 CMPs; they were more likely to find managerial ones (71%) than the ones relating to treatment (30%). Most identified CMPs were valid. Almost all causes of CMPs were plausible, but often too superficial to be of great value for directing remedial action. Audit teams suggested solutions, most of them promising ones, for 38% of the CMPs they had identified, but recorded their implementation only for a minority (8.5%).

**Conclusions:**

The importance of following-up and documenting the implementation of solutions should be stressed in future audit interventions. Tools facilitating the follow-up should be made available. Near-miss case reviews hold promise, but their effectiveness to improve the quality of care sustainably and on a large scale still needs to be established.

## Background

Maternal mortality in sub-Saharan Africa stagnates at a high level, its contribution to the global maternal death toll increasing from 23% to 52% between 1980 and 2008 [1]. Direct complications of pregnancy and childbirth contribute, in Africa, approximately 68% to this mortality [2]. Such complications usually require emergency obstetric care in district hospitals, where IV therapy, blood transfusion, general anaesthesia, caesarean section etc. are available [3]. Evidence suggests quality of care in hospitals often to be poor, and that women incur considerable delays before obtaining emergency treatment [4]. Reasons include lack of essential resources, sub-standard case management and unsatisfactory provider-patient interaction [5].

Audits may have potential to address case management problems (CMPs) in hospitals [6]. Audits come in various guises, but have in common that they seek to identify and analyse shortcomings systematically, and to design and implement interventions to prevent such shortcomings in the future [7]. They operate on the assumptions that shortcomings are learning opportunities and ought to be used as such, that health workers in general wish to perform well but need support for doing so, and that, except in cases of gross negligence or malevolence, encouragement and assistance are more effective than punishment.

In this paper we report the experience of introducing audits in hospitals in Benin, West Africa, and document the extent to which audit teams could identify CMPs, analyse their causes, agree on solutions and put these solutions into practice.

## Methods

### The audit intervention

In 1999 we introduced obstetric near-miss case reviews in five hospitals in Southern Benin. A near-miss case was defined as a woman suffering from a life-threatening complication during pregnancy or six weeks after delivery, whose “immediate survival is threatened and who survive by chance or because of the hospital care they receive” [8]. For each type of near-miss (uterine pre-rupture/rupture; obstetric haemorrhage; severe pre-eclampsia/eclampsia; sepsis; anaemia), a panel of national and international experts agreed on an operational definition (Table 1). In the absence of appropriate national guidelines, and with the relevant WHO manual [9] not yet published, the project developed guidelines on the management of major complications, and distributed them to participating hospitals.

**Table 1:** Definition of near-miss complications

**Obstructed labour**	**All cases of ruptured uterus and uterine retraction ring**
Obstetric haemorrhage	Obstetric haemorrhage characterised by external OR concealed bleeding at any time during pregnancy OR at delivery OR after delivery,
	resulting in AT LEAST ONE of the following events:
	· shock
	· hysterectomy
	· blood transfusion
Infections	Infections in pregnancy OR after recent pregnancy with:
	· hyperthermia OR hypothermia OR clear obstetric source of infections
	· AND AT LEAST ONE of the following:
	o systolic blood pressure < 80 mm HG
	o confusion or restlessness
	o jaundice
	o oliguria < 100 ml during any 4 hour period
Hypertensive disorders in pregnancy	Hypertensive disorders in pregnancy are characterized as follows:
	*first case definition*
	· hypertension defined as blood pressure of > 140/90 mmHg OR a rise in systolic blood pressure by > 30 mmHg OR a rise in diastolic blood pressure by >15 mmHg
	· AND AT LEAST ONE of the following signs:
	o convulsion
	o coma
	o jaundice
	o pulmonary oedema
	o severe oliguria
	o massive proteinuria
	o impending eclampsia, defined as AT LEAST TWO of the following signs: visual disturbance OR severe headache OR epigastric pain
	*second case definition:*
	· convulsions
	· AND AT LEAST ONE of the following signs
	o anti hypertensive treatment
	o massive proteinuria
	o generalised oedema
Anaemia	Anaemia is characterised as follows:
	· haemoglobin ≤ 5g/dl
	· OR mucosa very pallid AND at least one of the following symptoms:
	o breathlessness
	o oedema of legs
	o generalised oedema
	· OR, in the absence of haemorrhage:
	blood transfusion OR iv iron injections

In each hospital a core audit team was constituted, consisting of a senior physician involved in obstetric care and a senior midwife; both received a financial incentive for assisting the research activities. Staff members of maternity units and other relevant hospital departments (laboratory, pharmacy, anaesthesiology, intensive care, hospital administration) and from referring health centres were invited to attend audit meetings on a case-by-case basis, and received a small amount for transport and refreshments. Audit teams received an initial five days training on objectives and conduct of audits, and a three day refresher training after 12 months. All hospitals received a small financial contribution for their running costs.

Audit teams in each hospital were requested to identify near-miss cases continuously, choosing one per month for discussion during the monthly audit meeting. We advised the teams to choose particularly informative cases, making sure that the scope of major pathologies was represented over time. A staff member prepared a summary of the selected case’s management on the basis of clinical records. The hospital’s social worker conducted an in-depth interview with the woman, preferably at home, sometimes involving relatives, to obtain additional information from the woman’s perspective, possibly not apparent from clinical records.

The audit meeting was chaired by a member of the core audit team. Participants were reminded of the meeting’s objectives. The staff member who had prepared the case summary presented the case, and the social worker reported on the interview with the woman and her relatives. Subsequently the participants were invited to identify and discuss the positive aspects of the case management, and the CMPs. The discussion followed the course of events chronologically: from before admission to hospital to admission, diagnosis finding, near-miss treatment, further case management and discharge (“gate-to-gate” approach) [8]. Once all CMPs were thought to be identified, participants were invited to identify the cause which led to the CMP, and a solution which could prevent a similar CMP in the future. Audit teams were encouraged to discuss CMPs, causes and solutions that were within their sphere of influence. For each solution, teams were asked to nominate a staff member responsible for implementing it, to report during the next audit meeting whether this had been successful, and if not, to re-discuss the CMP. A secretary took notes and wrote structured minutes, recording positive aspects, CMPs, causes, solutions and responsibilities.

The type of audit we implemented could be labelled “hospital-based multi-professional patient-centred near-miss case review”. Choosing this type of audit was based on the following assumptions: “case review” - discussing authentic examples of severe obstetric complications interests and engages health workers; “hospital-based” - having the hospital teams carry out the audit themselves enhances their sense of ownership and their willingness to make an effort in implementing solutions; “multi-professional” - involving staff from different professions improves communication between them and thus the quality of care; “patient-centred” - interviewing women provides additional information and an opportunity to confront professionals with women’s experiences and views, a possible eye opener for the humane dimension of emergency obstetric care; “near-miss” - discussing complications that have been survived includes, by definition, positive aspects, thus facilitates frank discussions and bears less risk for depression or unhelpful attribution of blame. Since near-miss cases occur much more often than maternal deaths, reviewing them offers learning opportunities for staff more frequently.

### Benin’s health system

Benin is a francophone West African country with a population of about 6 million. In 1999, the maternal mortality ratio was estimated at 498 maternal deaths per 100.000 live births [10]. Benin was divided into 34 health districts, of which 28 had a public or religious district hospital, in addition to six departmental and one national hospital. Specialised health professionals were few (1 obstetrician per 36,162, 1 midwife per 17,846 women in reproductive age) and concentrated in large cities. The use of maternal services was high: 78% of women delivered in a health facility. The high use of services combined with high maternal mortality suggests problems with the quality of care.

### Participating hospitals

The five participating hospitals vary in size, ownership and position in the health system pyramid (Table 2): four government and one private non-profit hospitals; one national, two regional and two district hospitals; 1,000 to 6,000 deliveries per year, 4 to 14 midwives per 1000 deliveries, and deliveries by Caesarean section between 15% and 33%. The incidence of maternal near-miss morbidity and maternal mortality did not vary much across Hospitals 1, 2, 4 and 5 (7.6% to 10.4%, 700 to 1200 per 100,000 live births, respectively). The exception was Hospital 3, a regional referral hospital where the near-miss incidence reached 22.9% and the maternal mortality ratio 3,200 per 100,000 live births.

**Table 2:** Characteristics of hospitals

**Characteristic; N=5**	**Hospital 1**	**Hospital 2**	**Hospital 3**	**Hospital 4**	**Hospital 5**
Level	National/Teaching	Regional	Regional	District	District
Ownership	Government	Government	Government	Government	Private non-profit
Maternity beds (per 1000 deliveries/year)	78 (19.5)	200 (33.3)	73 (29.2)	23 (23.0)	14 (14.0)
Physicians covering maternity (per 1000 deliveries/year)	19 (4.8)	24 (4,0)	7 (2.8)	2 (2.0)	2 (2.0)
Midwives (per 1000 deliveries/year)	42 (10.5)	42 (7.0)	35 (14.0)	4 (4.0)	14 (14.0)
1 to 1 monitoring for severely ill patients*	Yes	Yes	Yes	No	No
Availability of 0 Rh neg blood	Sometimes	Sometimes	Sometimes	Sometimes	Sometimes
Availability of Caesarean sections	Always	Always	Always	Usually	Usually
Deliveries per year	4000	6000	2500	1000	1000
Caesarean section %	32%	24%	33%	21%	15%
Availability of emergency drugs	Usually	Usually	Usually	Usually	Usually
Maternal near-miss cases per 100 deliveries	10.4	7.6	22.9	8.1	8.9
Maternal mortality ratio per 100,000 life births	1200	900	3200	700	?
Number of audits^#^	16	17	15	12	7
Proportion (percentage) of audits moderated by physician^&^	13/13 (100.0%)	1/16 (6.3%)	4/9 (44.4%)	6/8 (75%)	2/4 (50%)

*: Maternity has cubicle to provide 1 to 1 monitoring for severely ill patients.

^#^: Only audits for which case summary and audit minutes were available; N=67.

^&^: Audits moderated by midwives otherwise; N=50 because of missing data.

Hospitals performed between 7 and 17 audits between October 1999 and March 2001. Due to different local policies the percentage of audits moderated by a physician as opposed to a midwife varied from 6.3% to 100%.

### Audit process evaluation

To carry out an audit, various documents were established: participants’ list, case summary with extracts from clinical records, summaries of the women’s interviews, and structured minutes of the audit meeting. For research, verbatim minutes were produced from audio-tapes of women’s interviews, and audit meetings were observed by social scientists, who did not intervene in the discussions but gave feedback at the end of the meeting and produced reports on their observations. Information was extracted from all these documents, and coded based on criteria as they appeared in the documents. Original clinical records were not available. The extracted information included: women’s age; obstetric history; date and time of the near-miss onset, admission, obstetric interventions, discharge, audit; type of referral and transport; type of near-miss complication and underlying cause; type of obstetric interventions; neonatal outcome if appropriate; woman’s satisfaction; number and profession of audit participants; and difficulties observed during the audit meeting.

Two external assessors (MB and TD, physician and obstetrician, respectively, both with experience in practising obstetrics in West Africa) used project guidelines, WHO guidelines [11] and occasionally a textbook [12] to decide what constituted a CMP. They independently identified CMPs from case summaries and women’s interview minutes. They extracted from minutes of the audit meetings the CMPs identified by the audit teams, assessed to what extent they were valid CMPs, and identified CMPs missed by the audit teams. Some clinical practices consistently deviated from guidelines without this ever being discussed by audit teams. It turned out that audit teams were unaware of the clinical standard, and could not be expected to identify and discuss these particular CMPs (Table 3); missing such CMPs was therefore “excused”. A complication caused or aggravated by inappropriate health seeking behaviour was not eligible as CMP, unless it happened after admission to hospital and staff’s failure to communicate effectively had apparently contributed to this behaviour. We categorised CMPs by type of CMP (diagnostic procedure missing, inadequate drug dosage, woman not informed etc.), by the timing or “gate-to-gate” phase in which they occurred, and by severity (i.e. the clinical significance for maternal survival or avoidance of long-term maternal morbidity). Categories for severity were “minor” (not directly relevant, e.g. dirty showers), “intermediate” (relevant, e.g. scarred uterus not diagnosed at admission, antibiotics underdosed) and “major” (immediate danger, e.g. failure to diagnose transverse lie, no rapid volume replacement for haemorrhagic shock). In case of disagreement, the assessors sought and mostly achieved consensus.

**Table 3:** Case management problems presumably due to standards unknown to audit teams*

	
***General***	No antibiotic prophylactic pre- or intra-operative
	Presence of foetal heart action confirmed without specifying frequency
***Pre-eclampsia, Eclampsia***	Using diazepam instead of magnesium sulphate to prevent or treat convulsions
	Using clonidine or methyldopa (or, rarely, nifedipine) as 1^st^ line antihypertensive instead of hydralazine
	No hourly auscultation of lung bases in severe pre-eclampsia and eclampsia
	Coagulopathy not routinely ruled out, no clotting test performed
***Haemorrhage***	Using colloids instead of crystalloids for volume replacement

*: These CMPs were observed in all relevant patients, never discussed by audit teams and therefore excluded from further analysis under the assumption that teams were not aware of the relevant standards.

From the minutes of the audit meetings one author (MB) assessed the performance of the audit team by constructing a score based on marks for the following achievements: 20 marks for ascertaining a valid CMP, regardless of whether the external assessors had identified the CMP from case summary or interview minutes; 10 marks each for analysing the causes of this CMP in a plausible way, in appropriate depth (profound enough to facilitate finding a solution *vs.* too superficial to be helpful), and taking into account their realm of influence; 10 marks each for agreeing on solutions that were plausible, addressed the problem in appropriate depth, and were within reach; 10 marks for assigning responsibility to one team member; 10 marks for reportedly having put the solution into practice or re-discussing it on the next audit meeting. The audit performance score was then computed per audit meeting as a percentage of achieved marks over achievable marks, thus taking into account that the achievable marks varied with the number of valid CMPs per meeting.

We analysed the performance of audit teams by individual CMP and by audit meeting. When analysing the performance by individual CMP, we either insisted on a valid analysis of the cause, or were satisfied when an appropriate solution was proposed regardless whether a valid cause had been identified. We used Chi^2^-tests or Fisher exact tests for comparing proportions as appropriate, and F-tests for comparing means. We used multiple linear regression and t-tests to verify the independence of determinants of audit team performance.

### Ethics approval

The ethics committee of the London School of Hygiene and Tropical Medicine approved the study (12 November 1997, no. 470), and the Ministry of Health in Benin gave permission to carry it out. Hospital managers were approached and agreed to let their hospitals participate in the study.

## Results

### Patients, complications, treatments

During the study period, 77 near-miss cases were reviewed. The analysis is restricted to 67 cases, for which case summary and audit minutes could be retrieved. The age of these women ranged from 16 to 46 (median 28); 18% were primigravidae, 18% grand multipara.

Seventy percent of the women had entered the hospital as emergency, either referred by another health facility (56%) or on their own initiative (14%, Table 4). The most important emergency transport means were private cars (59%), only 16% arrived by ambulance. The most common near-miss complications were haemorrhage (42%), followed by hypertensive disorders (25%), obstructed labour (19%), and infections (9%). Two thirds of the patients received whole blood transfusions, one third colloid fluids (Table 5). Fourteen out of 17 patients with near-miss hypertensive disorders received anticonvulsive drugs, 16 antihypertensive drugs. Of 41 women not delivered when the near-miss situation began, 63% were delivered by caesarean section or laparatomy, none by ventouse or forceps. 16% of all women had a hysterectomy. Fifty-three percent of the children were born alive. A third was born healthy, another third showed signs of intrapartum suffering leading to neonatal asphyxia (17%) or resulting in fresh stillbirth (15%).

**Table 4:** Characteristics of obstetric complications

**Characteristic; N=67**				
Type of referral; N=66*	***n***		***%***	
emergency referral by health facility	37		56%	
emergency self-referral	9		14%	
*subtotal: any emergency admission*	*46*		*70%*	
elective referral by health facility	14		21%	
elective self-referral	6		9%	
Type of emergency transport to hospital; N=37*	***Emergency referral***	***Emergency self-referral***	***Total***	
			***n***	***%***
ambulance	5	1	6	16%
private car	19	3	22	59%
motorbike	7	1	8	22%
on foot	0	1	1	3%
***Type of near-miss complication***	***n***		***%***	
rupture of extrauterine gravidity	6		9%	
other antepartum haemorrhage	10		15%	
postpartum haemorrhage	10		15%	
intra-/postoperative haemorrhage	2		3%	
*subtotal: any NM haemorrhage*	*28*		*42%*	
prae-eclampsia	5		7%	
eclampsia	12		18%	
*subtotal: any NM hypertensive disorder*	*17*		*25%*	
obstructed labour without uterine rupture	4		6%	
obstructed labour with uterine rupture	9		13%	
*subtotal: any NM obstructed labour*	*13*		*19%*	
postabortion infection/sepsis	4		6%	
postpartum infection/sepsis	2		3%	
*subtotal: any NM infection/sepsis*	*6*		*9%*	
NM anaemia	3		4%	

*N smaller than expected due to missing data.

**Table 5:** Management of obstetric complications

***Type of treatment: transfusion, infusion***	***Haemorrhage N=28***	***Uterine rupture N=9***	***Anaemia N=3***	***Other N=27***	***Total N=67***
whole blood	26/28	7/9	3/3	10/27	46/67 (69%)
plasma	8/28	3/9	0/3	2/27	13/67 (19%)
colloids	16/28	3/9	1/3	4/27	24/67 (36%)
crystalloids	21/26*	7/8*	0/3	17/26*	45/63* (71%)
***Type of treatment: anticonvulsives, antihypertensives, diuretics***	***Pre-eclampsia N=5***	***Eclampsia N=12***	***Other N=50***		***Total N=67***
anticonvulsives	5/5	9/12	2/50		16/67 (24%)
antihypertensives	5/5	11/12	4/50		20/67 (31%)
diuretics	2/5	3/12	3/50		8/67 (12%)
***Type of treatment: delivery***	***Obstructed labour N=4***	***Uterine rupture N=9***	***Hypertensive disorders N=17***	***Other N=11***	***Total***^***$***^***N=41***
vaginal birth without instrument	0/4	0/9	7/17	8/11	15/41 (37%)
vaginal birth with ventouse or forceps	0/4	0/9	0/17	0/11	0/41 (0%)
caesarean section, laparatomy	4/4	9/9	10/17	3/11	26/41 (63%)
***Type of treatment: hysterectomy***	***Haemorrhage N=28***	***Uterine rupture N=9***	***Post-partum/-abortion infection N=6***	***Other N=24***	***Total N=67***
	5/28	3/9	3/6	0/24	11 (16%)
***Hospital births: Immediate neonatal outcome; N=47***^***£***^	***n (%)***				
live birth: healthy	17 (36%)				
live birth: asphyxia	8 (17%)				
*subtotal: live births*	*25 (53%)*				
stillbirth: mature, fresh	7 (15%)				
*of which at admission heartbeat*	*positive*		*negative*	*unknown*	
	*3*		*2*	*2*	
stillbirth: mature, macerated	8 (17%)				
stillbirth: malformations	1 (2%)				
stillbirth: immature	0 (0%)				
stillbirth: undifferentiated	6 (13%)				
*subtotal: stillbirths*	*22 (47%)*				

*N smaller than expected due to missing data.

^$^: excludes women who did not deliver, or who had delivered before the near-miss complication occurred.

^£^: excluding 2 children born at home, 3 born in a health centre prior to admission to hospital.

### Audits

Audits took place on average one month after admission (Table 6). On average, meetings lasted 1.5 hours and had 15 participants. A third of the participants were health workers not involved in the clinical management of the reviewed case. In virtually all meetings, physicians, midwives, nurses and social workers were present, while laboratory technicians participated in half, and administrative staff in a quarter of the audits. Half of the audits were chaired by a physician, the others by a midwife or nurse. Most case presentations were done by a midwife or nurse (85% *vs.* 15% by a doctor). Discussions were lively and in general followed the recommended steps. The most frequent problems observed during the audit meetings were heated arguments between participants (45%), poor moderation (39%), inattentive (25%) or dominant participants (19%); poor recall of case management details was rare (3%).

**Table 6:** Characteristics of audit meetings

***Characteristic; N=67***	***median (min, q1, q3, max)***	
Interval admission-audit; N=65	39 days (8, 21, 62, 139)	
Duration of meeting; N=48	158 min (45, 134, 175, 239)	
Number of participants per meeting; N=57	15 (6, 12, 18, 24)	
Number of invited absentees per meeting; N=39	3 (0, 2, 6, 15)	
Involvement of participants in management of audited case; N=35	Yes:	No:
	46%* (0,38,58,69)	38%* (0,15,48,77)
***Presence of:***	***n (%)***	
obstetrician/physician; N=58	56 (97%)	
midwife/nurse; N=57	56 (98%)	
anaesthetist; N=57	39 (68%)	
lab technician; N=57	29 (51%)	
social worker; N=57	52 (91%)	
administrators; N=57	13 (23%)	
researchers; N=43	38 (89%)	
***Roles in the audit meeting***	***physician/obstetrician***	***midwife/nurse***
Meeting moderated by…; N=50	26 (52%)	24 (48%)
Case presented by…; N=53	8 (15%)	45 (85%)

*: Percentages do not add up to 100% because for some participants this information was not available.

### Factors facilitating patients’ survival

The most important facilitators for women’s survival identified by audit teams were those linked to human resources (95%), with commitment, willingness and availability (73%) ranking before competence (67%) and good teamwork (9%). Financial resources came second (73%), with patients having funds available (57%) mentioned more often than hospital funds for destitute patients (16%); in three cases, staff reported having paid for patients from their own pockets. Material resources ranked third (54%) and included the availability of blood (12%) and drugs (10%) from emergency kits in the hospital (*trousse d’urgence*) or in pharmacies with 24/24 opening hours. Other facilitating factors were clinical guidelines (19%), timely referral (19%) and good collaboration with and understanding by the women’s families (16%).

### Case management problems

During 67 audit meetings, the audit teams identified 413 case management problems (CMPs), of which the external assessors found 376 (91.0%) to be valid; invalid CMPs included women not attending antenatal care, families not providing moral support to the woman, and health workers using outdated medical terminology for the diagnosis. As mentioned before, audit teams consistently missed certain CMPs, being presumably unaware of the relevant clinical standard; these CMPs were exempt from further analysis (Table 3). The external assessors identified 338 CMPs not detected by the audit teams and not exempt. Further analysis of CMPs is carried out on the total of 714 CMPs.

Problems in the treatment of near-miss complications contributed most to the total of 714 CMPs (35%, Table 7), with delayed treatment reported in 11%, necessary drugs not given in 5.5%, unnecessary drugs given in 3.9% and therapeutic procedures not carried out satisfactorily in 3.5% of CMPs. Problems relating to diagnosis or follow-up were identified in 24% of CMPs, with insufficient monitoring in 9.0% and unsatisfactory physical examination in 5.9% of CMPs. Managerial problems were reported in 19% of CMPs, with insufficient documentation in 7.7% and unsatisfactory financial arrangements in 2.7% of CMPs. Unsatisfactory interaction with the patient constituted 14% of the CMPs, with insufficient provision of information (6.6%) leading before lack of friendliness and moral support (4.6%).

**Table 7:** Types of case-management problems, in detail

**N=714**	**n**	**%**
**Case-management problems related to …**		
***referral to hospital***	***42***	***5.8***
referral delayed	16	2.2
referral pattern/destination wrong	3	0.4
transport not “medicalised”	23	3.2
***establishing the diagnosis/monitoring***	***170***	***23.9***
initial diagnosis delayed	22	3.1
examination unsatisfactory	42	5.9
history taking unsatisfactory	15	2.1
diagnosis wrong	20	2.8
diagnosis incomplete	7	1.0
monitoring insufficient	64	9.0
***treatment***	***250***	***35.0***
treatment delayed	77	10.8
drug missing	39	5.5
drug overdosed	4	0.6
drug underdosed	21	2.9
drug unnecessary	28	3.9
drug wrong/not ideal	12	1.7
procedure missing	21	2.9
procedure carried out unsatisfactorily	25	3.5
procedure wrong	11	1.5
other	12	1.7
***management of hospital***	***136***	***19.2***
lack of cleanliness	9	1.3
staff not available	7	1.0
documentation insufficient	55	7.7
information flow unsatisfactory	12	1.7
equipment, supplies insufficient	14	2.0
financial arrangements unsatisfactory	19	2.7
other	20	2.8
***interacting with the patient or her family***	***98***	***13.7***
insufficient provision of information to the patient	47	6.6
lack of friendliness and support for the patient	33	4.6
not achieving compliance of the patient	18	2.5
***other***	***18***	***2.6***
delay (unspecified)	4	0.6
other	14	2.0

Audit teams identified 53% of all 714 CMPs classified as valid by the external assessors (Table 8). Audit teams were particularly proficient in identifying CMPs related to referral (88%) and hospital management (71%), and weak in identifying shortcomings in treatment of the near-miss complication (30%, p<0.0005). The capability of audit teams to identify CMPs did not vary with severity of the CMP (p=0.3).

**Table 8:** Type, timing and severity of valid case management problems

**N=714**	**Identified by audit team**		**Not identified by audit team, but by external assessors**		**Total**	
	**n**	**row %**	**n**	**row %**	**n**	**column %**
***Type of case management problem Case-management problems related to***	***Chi***^***2***^***-test: p<0.0005***					
referral to hospital	37	88.1	5	11.9	42	5.9
establishing the diagnosis/monitoring	95	55.9	75	44.1	170	23.8
treatment	76	30.4	174	69.6	250	35.0
interacting with the patient or her family	58	59.2	40	40.8	98	13.7
management of hospital	97	71.3	39	28.7	136	19.1
Other type of case management problem	13	72.2	5	27.8	18	2.5
**Total**	**376**	**52.7**	**338**	**47.3**	**714**	**100.0**
***Timing of case management problem Case-management problem occurred***	***Chi***^***2***^***-test: p<0.0005***					
before admission	66	67.4	32	32.6	98	13.7
at admission	24	63.2	14	36.8	38	5.3
while establishing diagnosis	51	55.4	41	44.6	92	12.9
while providing emergency treatment	57	33.7	112	66.3	169	23.7
… while providing further treatment	47	42.0	65	58.0	112	15.7
at discharge	44	60.3	29	39.7	73	10.2
undetermined time	74	67.9	35	32.1	109	15.3
after admission, before NM*	13	56.5	10	43.5	23	3.2
**Total**	**376**	**52.7**	**338**	**47.3**	**714**	**100.0**
***Severity of the case management problem***	***N=705***^***#***^***; Chi***^***2***^***-test: p=0.3***					
minor: not directly relevan^&^	183	55.6	146	44.4	329	46.7
intermediate: some relevance^&^	144	50.5	141	49.5	285	40.4
major: immediate danger^&^	44	48.4	47	51.7	91	12.9
**Total**	**371**	**52.6**	**334**	**47.4**	**705**	**100.0**

*: concerns patients whose near-miss started after admission to the hospital.

^#^: excluding 9 CMPs for which assessors could not reach agreement on severity.

^&^: …for maternal survival or avoidance of long-term maternal morbidity.

Audit teams were able to attribute causes for 83% of 354 CMPs they had identified (Table 9). Most causes were considered to be plausible (98%); an implausible cause was, for instance, the lack of attending ANC in the first trimester as a cause for the uterine rupture following a face presentation. While about 40% of the plausible causes were judged to be profound, i.e. to touch on the root cause of the CMP, almost 60% of the causes were judged as too superficial to be helpful in identifying an appropriate solution; examples include causes like “staff is negligent, staff has an inappropriate attitude”. Hospital teams were assumed to have some control over most plausible causes (90%); causes beyond reach included the lack of an ambulance at another health facility, or the assertion that “patient lacks funds”. Less than a quarter of all 376 valid CMPs identified by the audit teams had their causes appropriately analysed (found to be plausible, profound and within reach). Identification of appropriate causes worked better for CMPs related to near-miss treatment and worse for CMPs related to establishing the diagnosis and interaction with the women and their families, but not significantly so (p=0.071); the causal analysis of CMPs became more appropriate with increasing severity (p=0.001) and passed the test in 41% of the most severe cases.

**Table 9:** Appropriateness of CMP cause identified by audit team, by type and severity of CMP

**Type of CMP**	**CMP identified?**		**Cause identified?**		**Cause plausible?**		**Cause profound?**		**Cause within reach?**		**Cause identified *and *plausible *and *profound *and *within reach?**	
***By type of CMP***	***Yes***	***p<0.0005***^***#***^	***Yes***	***p=0.085***	***Yes***	***p=0.013***	***Yes***	***p=0.016***	***Yes***	***p=0.009***	***Yes***	***p=0.071***
referral to hospital	37/42	88.1%	23/26*	88.5%	20/23	87.0%	9/20	45.0%	16/20	80.0%	8/37	21.6%
establishing the diagnosis	95/170	55.9%	84/94*	89.4%	83/84	98.8%	27/83	32.5%	73/83	88.0%	17/95	17.9%
treatment of complication	76/250	30.4%	61/72*	84.7%	58/61	95.1%	35/58	60.3%	47/58	81.0%	25/76	32.9%
interacting with the patient	58/98	59.2%	40/55*	72.7%	40/40	100.0%	14/40	35.0%	36/40	90.0%	10/58	17.2%
management of hospital	97/136	71.3%	73/94*	77.7%	73/73	100.0%	25/73	34.3%	72/73	98.6%	24/97	24.7%
other	13/18	72.2%	12/13	92.3%	12/12	100.0%	6/12	50.0%	12/12	100.0%	6/7	46.2%
**Total**	**376/714**	**52.7%**	293/354*	**82.8%**	**286/293**	**97.6%**	**116/286**	**40.6%**	**256/286**	**89.5%**	**90/376**	**23.9%**
***By severity of CMP***	***Yes***	***p=0.31***^***#***^	***Yes***	***p=0.009***	***Yes***	***p=0.57***	***Yes***	***p=0.055***	***Yes***	***p=0.25***	***Yes***	***p=0.001***
minor	183/329	55.6%	131/172*	76.2%	129/131	98.5%	43/129	33.3%	115/129	89.2%	30/183	16.4%
intermediate	144/285	50.5%	119/135*	88.2%	115/119	96.6%	51/115	44.4%	102/115	88.7%	40/144	27.8%
major	44/91	48.4%	39/43*	90.7%	38/39	97.4%	20/38	52.6%	35/38	92.1%	18/44	40.9%
**Total**	**371/705**^**&**^	**52.6%**	289/350*	**82.6%**	**282/289**	**97.6%**	**114/282**	**40.4%**	**252/282**	**89.4%**	**88/371**	**23.7%**

^#^: p-values derived from chi^2^-test; all other p-values derived from Fisher’s exact test.

* N smaller than expected due to missing data.

^&^ N smaller than expected due to disagreement among external assessors on severity of CMP.

Audit teams recorded their suggested solutions for 202 (54%) of the CMPs they had identified (Table 10). Almost two thirds (129/202, 64%, data not shown) related to the way work was organised, most frequently by improving clinical documentation (12%), interaction with patients (11%), feed-back provided to referring health facilities (7%), and collaboration within the obstetrics team and with other hospital services (5%). While 18% of the solutions called for additional resources, mainly for training (8%) or improved availability of consumables (5%), only 16% of the solutions directly required changes in the medical procedures, for examination and monitoring (12%) or treatment (4%).

**Table 10:** Appropriateness of CMP solution suggested by audit team, by type and severity of CMP

**Type of CMP**	**CMP identified?**		**Solution identified?**		**Solution plausible?**		**Solution profound?**		**Solution within reach?**		**Solution identified *and *plausible *and *profound *and *within reach?**		**Follow-up: solution put in practice?**	
***By type of CMP***	***Yes***	***p<0.0005***^***#***^	***Yes***	***p=0.006***	***Yes***	***p=0.234***	***Yes***	***p=0.033***	***Yes***	***p=0.540***	***Yes***	***p=0.002***	***Yes***	***p=0.38***
referral to hospital	37/42	88.1%	13/37	35.1%	13/13	100.0%	13/13	100.0%	13/13	100.0%	13/37	35.1%	3/8*	37.5%
establishing the diagnosis	95/170	55.9%	48/95	50.5%	45/48	93.75%	29/45	64.4%	44/45	97.8%	28/95	29.5%	7/24*	29.2%
treatment of complication	76/250	30.4%	39/76	51.3%	37/39	94.87%	27/37	73.0%	36/37	97.3%	26/76	34.2%	12/25*	48.0%
interacting with the patient	58/98	59.2%	28/58	48.3%	28/28	100.0%	20/28	71.4%	28/28	100.0%	20/58	34.5%	5/7*	71.4%
management of hospital	97/136	71.3%	63/97	65.0%	63/63	100.0%	45/63	71.4%	63/63	100.0%	45/97	46.4%	14/30*	46.7%
other	13/18	72.2%	11/13	84.6%	11/11	100.0%	11/11	100.0%	11/11	100.0%	11/13	84.6%	2/3*	66.7%
**Total**	**376/714**	**52.7%**	**202/376**	**53.7%**	**197/202**	**97.5%**	**145/197**	**73.6%**	**195/197**	**99.0%**	**143/376**	**38.0%**	43/97*	**44.3%**
***By severity of CMP***	***Yes***	***p=0.31***^**#**^	***Yes***	***p=0.086***	***Yes***	***p=1.0***	***Yes***	***p=0.31***	***Yes***	***p=0.14***	***Yes***	***p=0.078***	***Yes***	***p=0.21***
minor	183/329*	55.6%	89/183	48.6%	87/89	97.8%	62/87	71.3%	87/87	100.0%	62/183	33.9%	21/39*	53.9%
intermediate	144/285*	50.5%	81/144	56.3%	79/81	97.5%	57/79	72.2%	78/79	98.7%	56/144	38.9%	16/46*	34.8%
major	44/91*	48.4%	29/44	65.9%	28/29	96.6%	24/28	85.7%	27/28	96.4%	23/44	52.3%	6/12	50%
**Total**	**371/705**	**52.6%**	**199/371**	**53.6%**	**194/199**	**97.5%**	**143/194**	**73.7%**	**192/194**	**99.0%**	**141/371**	**38.0%**	43/97*	**44.3%**

^#^ p-values derived from chi^2^-test; all other p-values derived from Fisher’s exact test.

* N smaller than expected because due to missing data, or disagreement among external assessors on severity of CMP.

Almost all solutions were plausible (Table 10). Three quarters of the 197 plausible solutions were judged to be profound; solutions that failed this test included those specifying an objective but not how it could be reached: “improve communication with laboratory”, “respect treatment protocol” etc. Virtually all 197 plausible solutions were within the reach of the hospital teams who ran the audits. In total, for 38% of the 376 valid CMPs identified by the audit team appropriate solutions were proposed. Audit teams performed better in proposing appropriate solutions for managerial CMPs and worse for CMPs relating to the diagnosis (p=0.002). Audit teams were also better, albeit not significantly so (p=0.078), in proposing appropriate solutions for the most severe CMPs (52%).

Audit teams recorded having identified a staff member as responsible to implement the solution for 37% of all solutions; this proportion did not vary by type of CMP (p=0.5) nor by severity (p=0.6). We have found documentation on whether or not solutions have been implemented for only 97 of 202 CMPs with proposed solutions (48%). For 44% of these, solutions have been reported to have been implemented, without variation between types and severity of CMP (Table 10).

**Figure 1:**
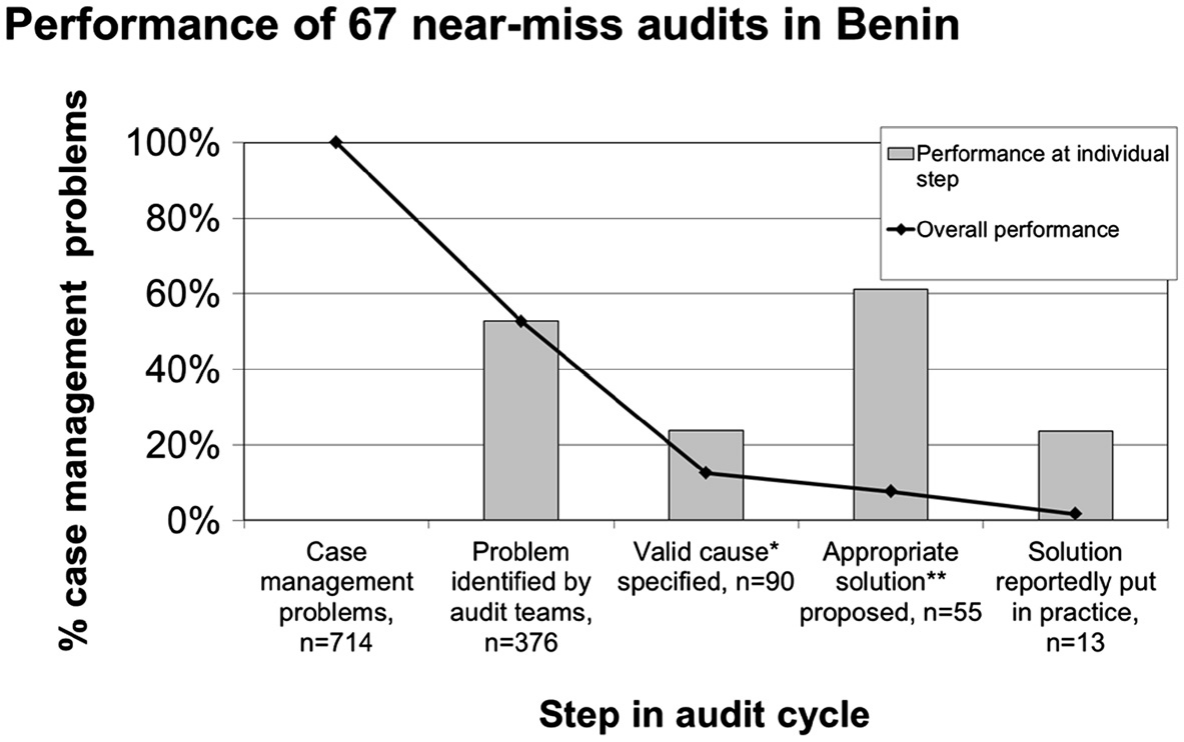
Performance of near-miss audits, requiring that a valid cause* has been identified for each CMP. *For a cause to be valid it had to be plausible, profound and within reach of the audit team. **For a solution to be appropriate it had to be plausible, profound and within reach of the audit team.

A comprehensive definition for handling a CMP overall appropriately requires that the CMP is recognised as such, *and* a valid cause identified, *and* an appropriate solution proposed *and* implemented by the audit team. For a total of 714 CMPs we found evidence in 376 (53%) that they were recognised, in 90 CMPs (13%) that valid causes had also been identified, in 55 CMPs (7.7%) that also appropriate solutions had been proposed and in 13 CMPs (1.8%) that these solutions had been implemented (Figure 1). Using only the CMPs recognised by audit teams as denominator, the percentage of CMPs with valid causes is 25%, and of CMPs for which solutions also have been proposed and implemented is 15% and 3.5%, respectively.

**Figure 2:**
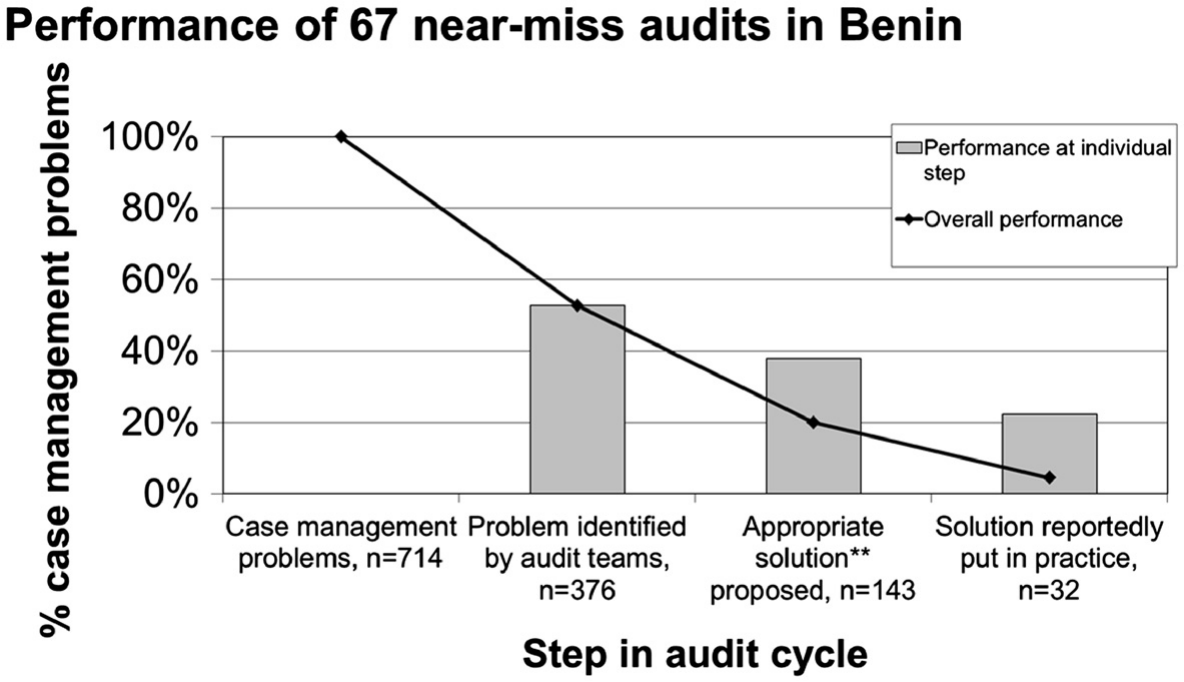
**Performance of near-miss audits, ****not** requiring that a valid cause* has been identified for each CMP. *For a cause to be valid it had to be plausible, profound and within reach of the audit team. **For a solution to be appropriate it had to be plausible, profound and within reach of the audit team.

A less challenging definition for overall appropriate handling requires that a CMP is recognised by the audit team, *and* an appropriate solution proposed *and* implemented regardless whether or not valid causes have been identified. Following this definition, for a total of 714 CMPs we found evidence in 143 CMPs (20%) that appropriate solutions have been proposed, and in 32 CMPs implemented (4.5%, Figure 2). Making the optimistic assumption that the proportion of implemented solutions (44%) is the same in CMPs with and without documentation on implementation, the proportion of CMPs for which an appropriate solution has supposedly been implemented increases to 10%. Taking only those CMPs recognised by the audit teams as denominator, the percentages for CMPs with appropriate solutions proposed and implemented are 38% and 8.5%, respectively. Making the same optimistic assumption that the proportion of CMPs with an implemented solution is identical for CMPs with and without documentation about implementation, the proportion of recognised CMPs with a supposedly implemented solution amounts to 19%.

We found the performance of audit teams to vary between hospitals (p=0.028, Table 11), but not by type of near-miss (p=0.7), nor that it improved over time (p=0.5). Teams moderated by a physician performed significantly better than those moderated by a midwife (p=0.030). This association persisted in multiple linear regression after controlling for type of near-miss and project maturation (*t*-test, p=0.028), but not when the hospital ID was added to the model instead (*t*-test, p=0.6). The effect of the moderator’s profession can therefore not be disentangled from other characteristics of the hospital.

**Table 11:** Determinants of the performance of audit teams

	**N, n**	**Mean performance score**	**SD**	**F-test, p-value**
***Location***	***67***	***-***	***-***	0.028*
National government hospital 1	16	32.6	11.0	
Regional government hospital 2	17	22.5	8.1	
Regional government hospital 3	15	27.4	10.6	
District government hospital 4	12	26.8	9.6	
District private non-profit hospital 5	7	21.3	6.1	
***Type of near-miss complication***	***67***	***-***	***-***	0.7*
NM haemorrhage	28	27.0	11.8	
NM hypertensive disorder	17	25.9	9.9	
NM obstructed labour	13	28.1	9.2	
NM infection/sepsis	6	27.7	5.6	
NM anaemia	3	18.8	4.4	
***Project maturity***	***67***	***-***	***-***	0.5*
First half of audits	33	27.5	10.2	
Second half of audits	34	25.9	10.1	
***Moderation of audit team***	***50***^***#***^	***-***	***-***	0.030*
by a midwife	24	23.4	9.1	
by a physician	26	29.7	10.7	

* Bartlett’s test for equal variance >> 0.05.

^#^ N<67because of missing data.

## Discussion

After introducing near-miss case reviews in five hospitals in Benin, we performed a process evaluation to assess to what extent audit teams were able perform near-miss case reviews according to the rules, that is, to identify CMPs and underlying causes, propose solutions, define responsibilities and document the implementation of solutions. Our study attempts to assess neither enhancements of quality of care, nor improvements of health outcomes.

Hospital teams readily adopted the audit intervention, organised meetings regularly and with appropriate frequency, and participated in large numbers – meetings were rather too large than too small, possibly resulting from incentives offered to participants, albeit modest (3 EUR). To preserve a frank exchange of views in large hospitals it may be necessary to restrict participation to those who were directly involved in the audited case’s management, and to brief the rest of the team on proposed solutions separately. The composition of participants was appropriately multi-professional. Midwives and nurses played crucial roles in the meetings by often moderating them and virtually always presenting the case summary. Not surprisingly, there was an important potential for conflict in these meetings, which underlines the importance of the moderators’ skills [13].

Audit participants acknowledged the overwhelming importance of health workers’ attitudes and competence for a positive patient outcome. Health workers are aware that available resources allow providing life saving obstetric care, if used by motivated and skilled health workers. The importance of financial arrangements for the poor and of the availability of blood and emergency drugs was acknowledged, which points to possible targets of interventions to improve the capability of Benin’s hospitals to respond to obstetric emergencies.

Most CMPs discussed by the audit teams merited auditing. Worrisome, however, is the high proportion of CMPs related to the treatment of complications (70%) that was missed by the audit teams. It appears that audit teams were more able or willing to discuss managerial shortcomings than medical ones. As most treatment decisions are either taken or approved by physicians, it is possible that the majority of participants did not feel competent or entitled to challenge these decisions. This may be an inherent limitation of internal case reviews when carried out in district hospitals with few physicians involved in emergency obstetric care; possibly the involvement of a higher level, e.g. the obstetrician from the regional referral hospital, could help, but one would have to look out for any threatening or intimidating effect this may have on the audit team. The lack of detailed evidence-based guidelines on the management of obstetric complications at the time of the study has probably contributed to the under-representation of therapeutic CMPs in the audit meetings. In the meanwhile WHO has published such guidelines [11] in several languages (e.g. Arabic, English, French, Indonesian, Russian and Spanish), so that case reviews should now use these or equivalent guidelines. The guidelines may also raise the awareness in Benin that certain practices (Table 3) are no longer in line with international recommendations. The emerging consensus on near-miss case definitions may help developing curricula on how to diagnose and manage life-threatening obstetric complications [14].

Many causes recorded by the audit teams appeared to be too superficial to be of value for identifying the most appropriate solution: to note that something has not been done because of “negligence” without analysing where motivational problems come from hardly points into the direction remedial action should take. It is possible that the discussion had more substance than what has been recorded in the minutes. In any case the “why? why?” root cause analysis described by Weeks *et al.*[15] may constitute an improvement over the audit practice described here: it involves questioning the CMP’s cause that comes first to mind in order to get to the root cause of the problem. It is encouraging, however, that audit teams in general accepted responsibility for the CMP, and rarely located its cause outside their sphere of influence.

Audit teams proposed solutions for only half of the CMPs they identified themselves. It is not clear whether this represents an oversight, a decision to focus on some CMPs and not to seek solutions for others, or a failed attempt to find and agree on solutions. In any case, the high proportion of CMPs identified but not tackled by the audit teams is worrying. As reported from neighbouring Burkina Faso [16], many solutions addressed organisational issues, fewer medical procedures, and very few treatment, possibly for the same reasons why audit teams missed many CMPs related to treatment in the first place. It is reassuring that most solutions were plausible, directed towards the CMP’s root cause, and within reach of the audit team; many did not appear to require significant additional resources.

A sobering finding is the low proportion of CMPs with implemented solutions. Solutions were at best implemented for 19.3% of the CMPs, while the proportion of CMPs for which implemented solutions have been documented was 8.5% only. We may have underestimated this proportion by not following up for long enough; in Burkina Faso it was found that it may take several months to implement a solution [16]. However, this alone is unlikely to explain the considerable gap between proposed and implemented solutions. Future audit trainings should underline the importance of implementation and follow-up. Tools should be used that facilitate the follow-up of agreed solutions, for instance an audit register with the columns “problem | why | why | solution proposed | responsible | deadline | follow-up”. If the follow-up reveals that solutions have not been implemented by the agreed deadline, the audit team needs to reconsider the course of action. Possibly, incentives should be tied to the implementation of solutions, and not to the participation in audit meetings. If action does not follow the talking, audits risk degenerating into a fruitless palaver which bears few prospects to improve quality of care.

The number of participating hospitals (N=5) was too small for a meaningful analysis of hospital factors determining the variable performance of audit teams. We have found that audit teams perform better if moderated by physicians, which may be due to their more profound medical knowledge and their greater authority, but we cannot rule out confounding by other hospital factors we were unable to elicit.

The variety of case reviews carried out in Benin included interviews with patients and their relatives, whose results were than reported back to the audit team. Whether these interviews had an overall engaging or disruptive effect on the discussions of the audit team remains unclear and merits further research. A considerable variation in quality, from rich to superficial, from encouraging and respectful to confrontational and judgemental, transpired from the interview transcripts. This demonstrates that training and supervising staff carrying out these interviews, e.g. social workers, is essential.

Another open question is whether near-miss case reviews should complement or replace maternal death reviews. The exclusion of maternal deaths from case reviews is likely to facilitate the audit process, as the question of guilt is less burning when the woman has survived. Certain shortcomings in case management, e.g. unacceptable delays, may only be recognisable from maternal deaths but not from near-miss cases; if maternal deaths concentrate during certain times of the day or the week this may point at underlying causes, which will be missed by excluding maternal deaths from review. Operational research should investigate whether experienced case review teams can cope with the additional challenge to review a maternal death and remain faithful to the principles of frankness and non-accusation.

Finally, the question of sustainability of audits arises. Often, while there is special funding, audits tend to be implemented and conducted, but without that funding audits are less likely to happen. Once the effectiveness of audits has been demonstrated hospital management, with the support of the Ministry of Health, should advocate that they are an internal quality improvement technique and as such part of the staff’s duty, and not an optional activity that needs to be paid extra. When Hutchinson *et al.* found that all hospitals participating in this study had stopped doing audits within two years after completing this feasibility study, lack of resources was one, but not the most prominent reason [17]. Support by Ministries of Health has increased recently, and many national plans now include audits of maternal deaths as a compulsory activity to improve quality of care.

### Limitations of the study

This study has several limitations. Most importantly, no primary data like clinical records or tape recordings of patient interviews or audit meetings were available; the data sources were limited to documents produced by health workers to prepare and document the audits. We are likely to have missed some CMPs if they have not transpired from case summaries or patient interview transcripts. We may also not have fully captured the audit teams’ reasoning on causes of and solutions for CMPs if it is not reflected in the audit minutes. We may have missed solutions that were implemented but not documented. Because of the difficulty to distinguish between shortcomings in the audits’ conduct and weaknesses in their documentation, their quantitative assessment should be interpreted with caution, but we have confidence in the principal patterns that have emerged.

The presence of researchers as observers, i.e. social scientists and epidemiologists, in many audit sessions may have positively influenced the way audits were conducted, but audits were still far from perfect and allowed many shortcomings to be identified.

It may be argued that the observation time was too short, and that audit teams would have learned to perform audits better over time. However, our data do not support this assumption, and we doubt that, without further training, audit teams would by themselves have been able to improve significantly.

Because of the purposive sampling of audit cases by the audit teams, selected cases do not constitute a representative sample of all near-miss cases treated in the participating hospitals. The audit teams may have chosen patients with particularly poor case management to maximise learning, or those who were relatively well looked after to limit embarrassment. No conclusion can be drawn on the relative frequency of type of near-miss, admission, CMP etc. in those hospitals. However, the validity of our analysis of how well the audit teams dealt with the cases they have selected is not compromised by this non-representativity of the sample.

We have not studied hospital environment or team dynamics in detail. It is therefore difficult to conclude to which extent the results are specific to these five hospitals or can be generalised to hospitals in similar settings. We have some confidence in the external validity of our results, as the participating hospitals represent various levels of the health system pyramid and both public and private sectors.

The data of this study are now 10–12 years old. However, we have continued working on the quality of obstetric care in this setting and believe that the results are still relevant for current efforts to implement audits.

## Conclusions

Quality assurance approaches based on near-miss morbidity remain highly topical [18]. Near-miss case reviews have the potential to improve the quality of managing life-threatening obstetric complications in Benin’s hospitals, a setting where resources are scarce and must be used with consideration. The reviews were popular with staff and carried out regularly, and the audit teams were able to analyse shortcomings and make suggestions as to how they can be avoided in future. While the principle of such reviews is deceptively simple, they are not a quick fix, and their implementation needs thorough training and follow-up. Ultimately, their capacity to improve the quality of care in settings similar to the one studied here still needs to be demonstrated – something the ongoing AUDOBEM trial (ISRCTN67206260) attempts to do.

## Abbreviations

CMP: Case Management Problem; WHO: World Health Organisation.

## Competing interests

Matthias BORCHERT is Principal Investigator of the AUDOBEM trial. Sourou GOUFODJI, Eusèbe ALIHONOU, Thérèse DELVAUX and Lydie KANHONOU are Co-Investigators of the AUDOBEM trial.

## Authors’ contributions

MB analysed and interpreted the data and drafted the article. SG, EA, JS and LK substantially contributed to the acquisition of data and revised the draft critically for content. TD substantially contributed to the analysis of the data and revised the draft critically for content. VF conceived and designed the study, substantially contributed to interpretation of the data and revised the draft critically for content. All authors read and approved the final manuscript.

## Pre-publication history

The pre-publication history for this paper can be accessed here:

http://www.biomedcentral.com/1471-2393/12/109/prepub
